# Autism spectrum disorder detection using diffusion tensor imaging and machine learning

**DOI:** 10.1371/journal.pdig.0001155

**Published:** 2025-12-23

**Authors:** Noel A. Cardenas-Hernandez, Marlen Perez-Diaz, Karla Batista García-Ramó, Maria del C. Valdés Hernández

**Affiliations:** 1 Department of Physics, Universidad Central “Marta Abreu” de Las Villas, Santa Clara, Cuba; 2 Department of Automatics, Universidad Central “Marta Abreu” de Las Villas, Santa Clara, Cuba; 3 Centre for Neuroscience Studies, Department of Medicine, Queen’s University, Kingston, Ontario, Canada; 4 Clinical Research Department, Center of Isotopes, Havana, Cuba; 5 Department of Neuroimaging Sciences, Centre for Clinical Brain Sciences, University of Edinburgh, Edinburgh, United Kingdom; North Carolina A&T State University: North Carolina Agricultural and Technical State University, UNITED STATES OF AMERICA

## Abstract

Autism spectrum disorder (ASD) is a neurological and developmental disorder that manifests in social and behavioral deficits. The onset of symptoms may begin in early childhood, but diagnosis is often subjective, and scores can vary between specialists. Several studies suggest that diffusion tensor imaging (DTI)-derived indicators of anisotropy in water diffusion at microstructural level could be biomarkers for this disorder. Emerging advances in neuroimaging and machine learning can provide a fast and objective alternative for its early diagnosis. We propose and evaluate a machine-learning (ML)-powered computer-aided diagnosis (CAD) system for the detection of ASD from DTI. For the development and validation of the system we used the ABIDE II database (n = 150). The system involves processing the raw DTI to obtain fractional anisotropy (FA), mean diffusivity (MD), radial diffusivity (RD) and axial diffusivity (AD) in 25 ASD-relevant regions of interest defined in the JHU ICBM-DTI-81 White-Matter Labeled Atlas to train a ML binary classifier. We evaluated the use of support vector machine (SVM) with various kernels and random forest (RF) optimized for computational efficiency. The best configuration, which used RF, had a sensitivity of 100%, accuracy of 95.65%, precision of 91.67%, and a specificity of 91.67%. An external test yielded 94.73% sensitivity, 97.37% accuracy, and 100% in precision and specificity. Results in this small sample show the generalization power of the best model, and the utility of carefully leveraging imaging information with clinical knowledge on relevant white matter regions commonly affected by ASD to design a CAD system for ASD.

## Author summary

Autism spectrum disorder (ASD) is a condition that starts manifesting in early years but which is difficult to diagnose due to similarities with other conditions like attention deficit hyperactivity disorder. Early diagnosis can make a real difference in the child’s ability to communicate and behave later. Various studies suggest that a modality of magnetic resonance imaging that allow scientists to study the brain connections and, with it, the tissue micro-structure, named diffusion tensor imaging (DTI) can be a good alternative for early diagnosis of this condition. We propose and evaluate a machine-learning (ML)-powered computer-aided diagnosis (CAD) system for the detection of ASD from DTI. We use DTI data from 150 contributors to the ABIDE II public database. From these data, we generated four indicators of brain microstructural integrity in different brain zones, guided by a relevant brain atlas. We used various configurations of two ML popular schemes for our tasks, tuned their parameters, and evaluated different combinations of the brain health indicators we derived from the DTI. We finally evaluated the system that produced the best results in an unseen data. All our evaluations suggest that the CAD system we propose can be useful and a good candidate to be considered for further application in clinical research and practice to diagnose ASD.

## Introduction

Autism spectrum disorder (ASD) is a neurological and developmental disorder that manifests in persistent deficits mainly in the following areas of social communication and interaction: social-emotional reciprocity; developing, understanding, and maintaining relationships; and nonverbal communication [1]. The prevalence of ASD is increasing globally, with a median prevalence of 100/10,000 (ranging from 1.09/10,000 to 436.0/10,000) individuals [2]. Research tracking the early development of children with ASD has revealed distinctive patterns that include difficulties in diverting visual attention from a stimulus, deficits in self-regulation ability, and impairments in the neurocognitive processing of social information in different degrees [3]. Early detection and specialized intervention are crucial for the development of skills and quality of life of people with ASD [4].

Currently, ASD diagnostic methods consist of collecting clinical information about the patient’s development and direct observation of their behaviour [5,6]. But ASD also co-occurs with other behavioral disorders like attention deficit hyperactivity disorder (ADHD) [7], dyslexia, dyspraxia, obsessive compulsive disorder (OCD), anxiety and depression [8], and comorbidities like epilepsy, and gastro-intestinal and sleep disorders among others [9] all which introduce complexity to its diagnosis. Taking into account that medical images offer vital information for accurate diagnoses and treatment monitoring, and that among imaging diagnostic modalities, magnetic resonance imaging (MRI) stands out for providing accurate, comprehensive and multiparametric information on brain anatomy and function non-invasively, it has emerged as a tool to support the earlier diagnosis of multiple diseases such as ASD.

Among the different MRI sequences, diffusion-tensor images (DTI) have been widely used to study the microstructural characteristics of the brain tissue [10] through a set of parameters that characterize the diffusivity of water in the brain. These are the axial diffusivity (AD), the fractional anisotropy (FA), the mean diffusivity (MD), and the radial diffusivity (RD), which have been applied to a large group of brain pathologies [11]. In ASD children and adolescents, most studies have found compromised white matter integrity, manifested in low FA and high MD (and RD) values in widespread areas of the white matter [12,13]. A study found decreased FA specifically in white matter tracts that connect brain areas associated with social functioning (i.e., fusiform gyrus, amygdala, and superior temporal gyrus) even after controlling for age and IQ [14]. It has also been reported increase in diffusivity and decrease in FA values in the corpus callosum [15–17]. Other studies have reported similar patterns in the longitudinal fascicles [15,17–19], the brainstem tracts (cerebral peduncle) [17], and projection fibers (anterior and posterior extremities of the internal capsule; anterior, posterior, and superior corona radiata; and posterior thalamic radiation) [17]. Compared to controls, individuals with ASD, ranging from children and adolescents [17] to adults [16], have been found to have lower AD in the regions of the corpus callosum [16,17], cerebral peduncle, sagittal stratum including the longitudinal fasciculus, cingulum, and several projection fibers (anterior and posterior branches of the internal capsule; retrolenticular internal capsule; anterior and superior corona radiata; external capsule) [17]. A study found the subgenual anterior cingulate cortex with increased MD values in ASD children compared to controls. For ASD detection, tensor asymmetry has also been analyzed [20–23], since it reveals information not captured by other features [24]. However, it is important to note that although most studies have found decreased FA in ASD, some studies have found increased FA [25], or a combination of increased and decreased FA in different white matter areas, in people with ASD compared to typically developing individuals [26]. These aspects are not yet fully clarified, so the research area is open.

Machine learning and more recently deep learning methods have been applied to automatically detect ASD from various indicators, being the most common derived from functional MRI, behavioral tests, behavioral electronic monitoring, and structural MRI [5]. To the best of our knowledge, only few studies have been conducted to propose a fully automatic computer-aided diagnosis (CAD) system for ASD diagnosis using solely DTI [20,21,27–29]. From these, three studies propose a support vector machine (SVM) classifier with a linear kernel as the best of the methods evaluated, with best accuracies being 71% [27], 75% [29], and 99% [20,21], and one [28] proposes a linear regression classifier (81% highest accuracy). Deep learning methods have also been evaluated with suboptimal results [20,21]. But the study reporting 99% accuracy does not report Precision (Sensitivity) and Recall (Specificity) and neither performed external testing, so it is not known if the results are reproducible or not in unseen data acquired in a different imaging system with a different MRI protocol.

Our contributions are two-fold:

1) We benchmark and document the performance of different configurations of the two most commonly used ML classifiers for ASD detection solely using DTI in large multicenter data.
2) The sensitivity and specificity values obtained by the two best models chosen for validation, this done in an external dataset, exceed 90%, being on the order of or exceeding those obtained by other state-of-the-art CAD systems developed for ASD, reviewed by Song et al. [30] and Bae et al. [31].

## Materials and methods

### Dataset

We use structural MRI (T1-weighted) and diffusion tensor imaging (DTI) scans of the Autism Brain Imaging Data Exchange (ABIDE-II) [32] publicly available database (https://fcon_1000.projects.nitrc.org/indi/abide/abide_II.html). From the 19 contributing centres, 5 provide DTI data in nifti-1 format from 319 individuals (180 with ASD diagnosis and 139 without ASD labelled as typical controls). We used data from three: New York University Langone Medical Centre (NYU samples 1 and 2), San Diego State University (SDSU), and Trinity Centre for Health Sciences (TCD), which have the *bval* (i.e., lists the *b-value* for each volume in the series) and *bvec* (i.e., lists the gradient direction, with one column per volume) files present. Our dataset comprises image and basic demographic data from 150 individuals, of which 87 have ASD diagnosis and 63 have not and are used as controls. Table 1 summarises the demographic characteristics of the samples included.

**Table 1:** Sample demographic characteristics.

Data set	NYU1 (n = 44)		NYU2 (n = 14)		SDSU (n = 54)		TCD (n = 38)	
Category	ASD	C	ASD	C	ASD	C	ASD	C
Total	24	20	14	0	30	24	19	19
Age range (years)	5.2 – 34.7	5.8 – 23.8	5.1 – 8.8	–	7.4 – 18.0	8.1 – 17.7	10.0 – 19.5	10.2 – 20.0
Sex	M, F	M, F	M, F	–	M, F	M, F	M	M
Handedness	1, 2, 3	1, 2, 3	1, 2, 3	–	1, 2, 3	1, 2, 3	2	2

Legend: Group data obtained from ABIDE II website. Handedness: 1-right handed, 2-left-handed, 3-ambidextrous.

All MRI data were acquired using 3T scanners from different manufacturers: SIEMENS MAGNETOM Allegra syngo MR 2004A in NYU, GE 3T MR750 with an 8-channel head coil in SDSU, and Philips Intera Achieva SENSE with head-8 coil in TCD. The DTI acquisition for these samples was: TE/TR = 78/5200 ms and 0.32 ms echo spacing for NYU1, TE/TR = 3.25/2530 and 7.4 ms echo spacing for NYU2, TE/TR = min/8500 ms for SDSU, and 79/20244 ms for TCD. NYU DTI acquisition has 64 directions, with voxel sizes 3.0 × 3.0 × 3.0 mm^3^ for sample 1 and 1.3 × 1.0 × 1.3 mm^3^ for sample 2. SDSU and TCD acquired DTI with 61 directions and voxel size 2.0 x 2.0 x 2.0 mm^3^. More details in the DTI acquisition at each centre can be found in the ABIDE II website (https://fcon_1000.projects.nitrc.org/indi/abide/abide_II.html).

### Preprocessing and feature extraction

**Fig 1:**
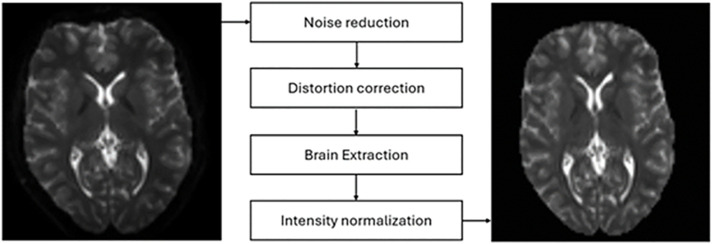
Pipeline used for Image Preprocessing.

The neuroimaging preprocessing followed a standard pipeline using MRtrix software package [33] (version 3.0.1, www.mrtrix.org/) and FSL (version 4.1) [34] (Fig 1). DTI data preprocessing included denoising, Gibbs ringing removal, corrections for image distortions, head motion, bias field, and image intensity normalization. Due to unavailability of an unweighted diffusion image with reversed phase-encoding Synb0-DISCO [35] was used to synthesize an unweighted diffusion image without susceptibility-induced distortion from the T1-weighted image.

**Fig 2:**
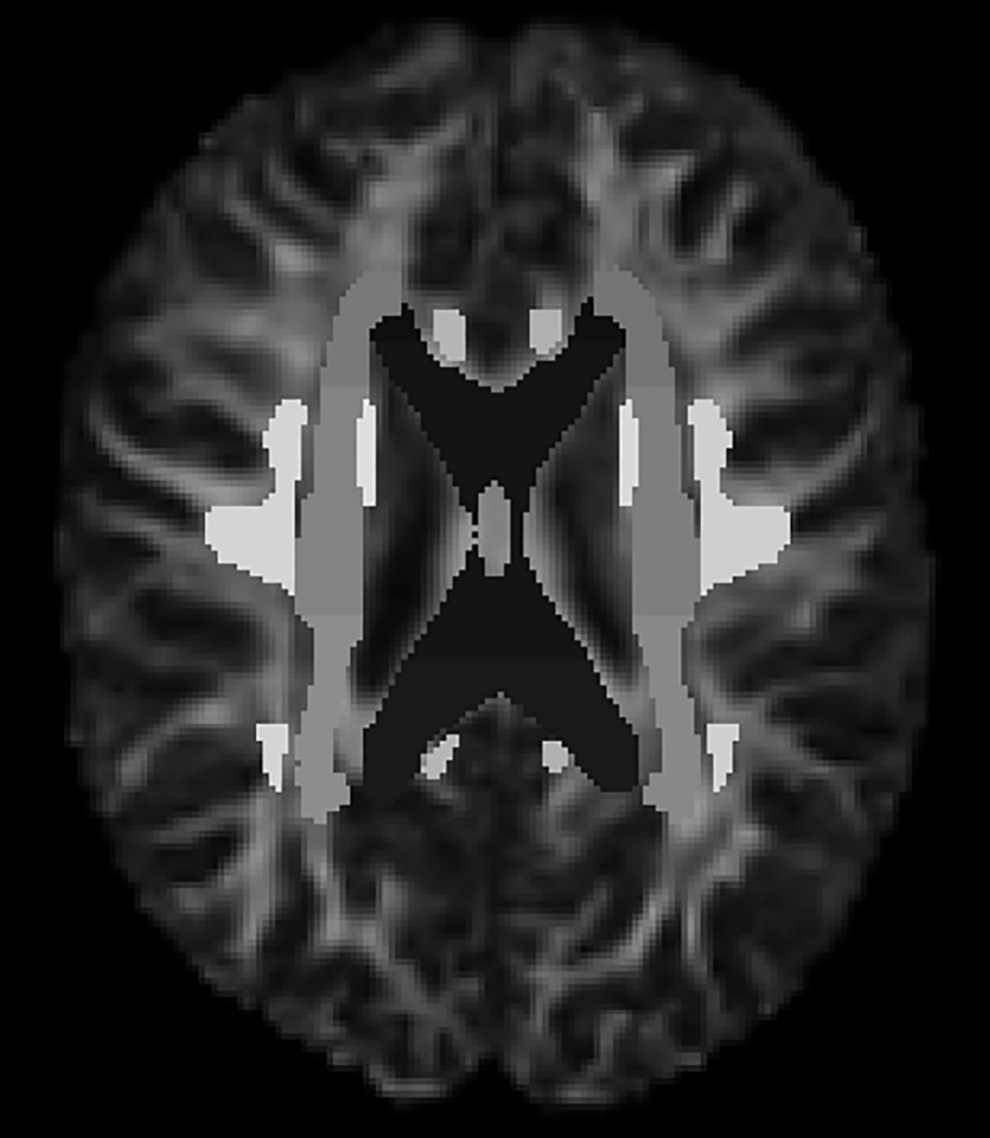
FA map and ICBM-DTI-81 atlas co-registered after using the flirt function.

The maps of axial, radial and diffusivity (AD, RD, MD), and fractional anisotropy (FA) were generated using FSL’s diffusion toolbox. An atlas-based segmentation approach was implemented co-registering the John Hopkins University ICBM-DTI-81 white matter (WM) atlas [36] which 48 white matter areas, to subject’s space (Fig 2). This allowed to represent each subject by four features per 48 areas.

Of the 48 regions defined in ICBM-DTI-81, the 25 that, according to the scientific literature, have shown ASD traits were selected based on the regions of interest analyzed in [15,17–19]. The selected regions are shown in Table 2. For each region defined in each image, we calculated the average values of FA, MD, AD, and RD. In this way, a total of 100 DTI features were calculated for each image. These features were organized into a CSV file to be used as input vector to the classifiers.

**Table 2:** Regions selected for feature extraction.

Region	Code ICBM-DTI-81
Corpus callosum	3,4,5
Cerebral peduncle	15,16
Anterior and posterior branches of the internal capsule	17,18,19,20
Retrolenticular internal capsule	21,22
Anterior and superior corona radiata	23,24,25,26
Inferior longitudinal fascicles	31,32
External capsule	33,34
Cingulum	35,36,37,38
Superior longitudinal fasciculi	41,42

### Machine learning classiﬁcation and evaluation of models

During this stage, we divided the data into two parts. Data derivatives from three sets (NYU1, NYU2 and SDSU) were used for training and validating the classifiers. Data derivatives from one set (TCD) were used for external testing. The classifiers were trained to classify patients with ASD and healthy individuals, but the input feature vector was constructed in two different ways: in one, all the calculated features were considered for classification, and in the other, the input only consisted of the FA and MD features, as these in combination have proven very useful for studying and revealing early changes in many neurological diseases including ASD [12,37]. The training set was randomly divided into two groups: 80% for training and 20% for validation. But we also accounted for key variables including ASD/control, age, sex and handedness, to ensure a balanced representation between groups as much as possible and minimise possible biases during training and testing. It is worth noting that ASD and controls were not 1-to-1 age- and sex-matched to utilize all the data available, but overall age distributions were not different between ASD and controls (see section Risk of bias and generalizability). Sensitivity, precision, accuracy, and specificity were used as metrics for model validation.

To implement the SVM classifier, we used the Python function *SVC* from the *Scikit-Learn* library. The parameters of this function are: kernel type (linear, radial, and polynomial of degrees 3, 5, and 7), regularization parameter *C* which controls the balance between the complexity of the decision boundary and the accuracy in classifying the training data, and the randomness parameter *random_state*. We used the default value *C* = 1, which implied a balanced configuration in the model implementation, and to ensure consistent behaviour the *random_state* was set up as null, which implied the model used random seeds in each run. To obtain the best fit for this model, several trial-and-error tests were performed, varying the kernel types used, and the best one was chosen for the test data. The trial-and-error strategy was guided by values reported in the literature, software documentation (https://scikit-learn.org/stable/modules/generated/sklearn.svm.SVC.html), and by empirical observations obtained in preliminary experiments, which facilitated the identification of adequate ranges for the classifiers, while allowing the viability of the study with limited computational resources.

To implement the Random Forest models, we used the *RandomForestClassifier* function from the *Scikit-Learn* library. The *n_estimators* parameter controls the number of trees created in the forest. The default values for the parameters that control tree size (e.g., *max_depth*, *min_samples_leaf*) were adjusted to reduce memory consumption, complexity, and tree size. *random_state* was set to none to obtain a consistent model during tuning. Multiple trial-and-error tests were performed on the *n_estimators* (i.e., 20, 50, and 100) and *max_depth* (i.e., none and 10) parameters.

All processing was carried out in a workstation with the following specifications: CPU: Intel Core i7-8700 3.2 GHz, graphic card NVIDIA GeForce RTX 3070 8 GB GDDR6, RAM 32 GB (2 x 16 GB) DDR4 3200MHz, base plaque MSI B365M PRO-VH, storage: SSD 128GB M.2 SATA + HDD 1TB (7200RPM).

Other Python libraries used, in addition to *Scikit-learn* for the ML algorithms, were *Nibabel* for the manipulation of nifti-1 files, *Numpy* for the manipulation of matrices and arrays, and *Matplotlib* and *Seaborn* for data visualisation and graphs. Statistical analyses of the results (i.e., descriptive statistics and group comparisons) were performed using the Statistical Package for the Social Sciences (SPSS) software. Post-hoc evaluations were performed using MATLAB R2023a. A significance level of 0.05 or less was considered a statistically significant result from any statistical test.

## Results

**Fig 3:**
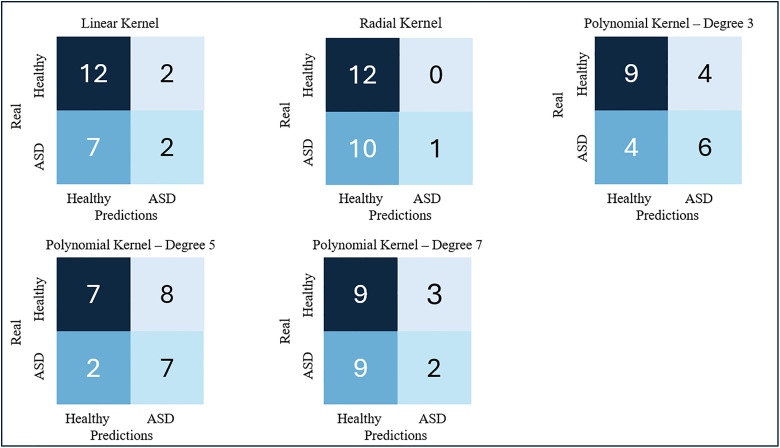
SVM results for a feature vector composed on the four DTI metrics: FA, MD, RD, and AD.

**Fig 4:**
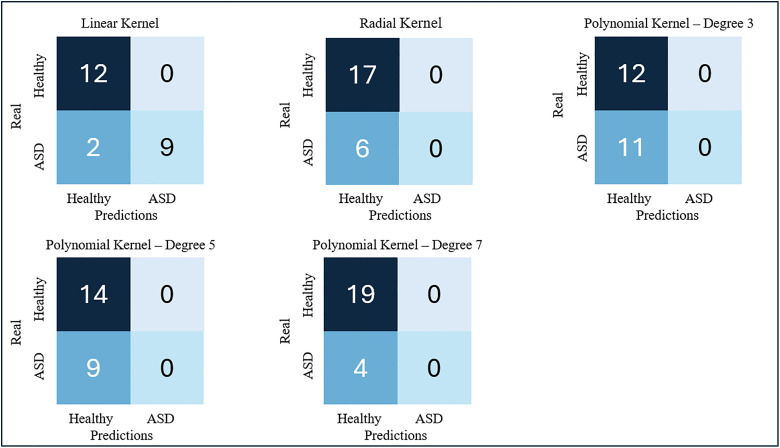
SVM results for a feature vector composed on two DTI metrics: FA and MD.

The SVM classifier for various kernel types yielded the results shown in Figs 3 and 4, first for a feature vector composed of the four computed DTI metrics (FA, MD, RD and AD), and then for two features (FA and MD).

**Fig 5:**
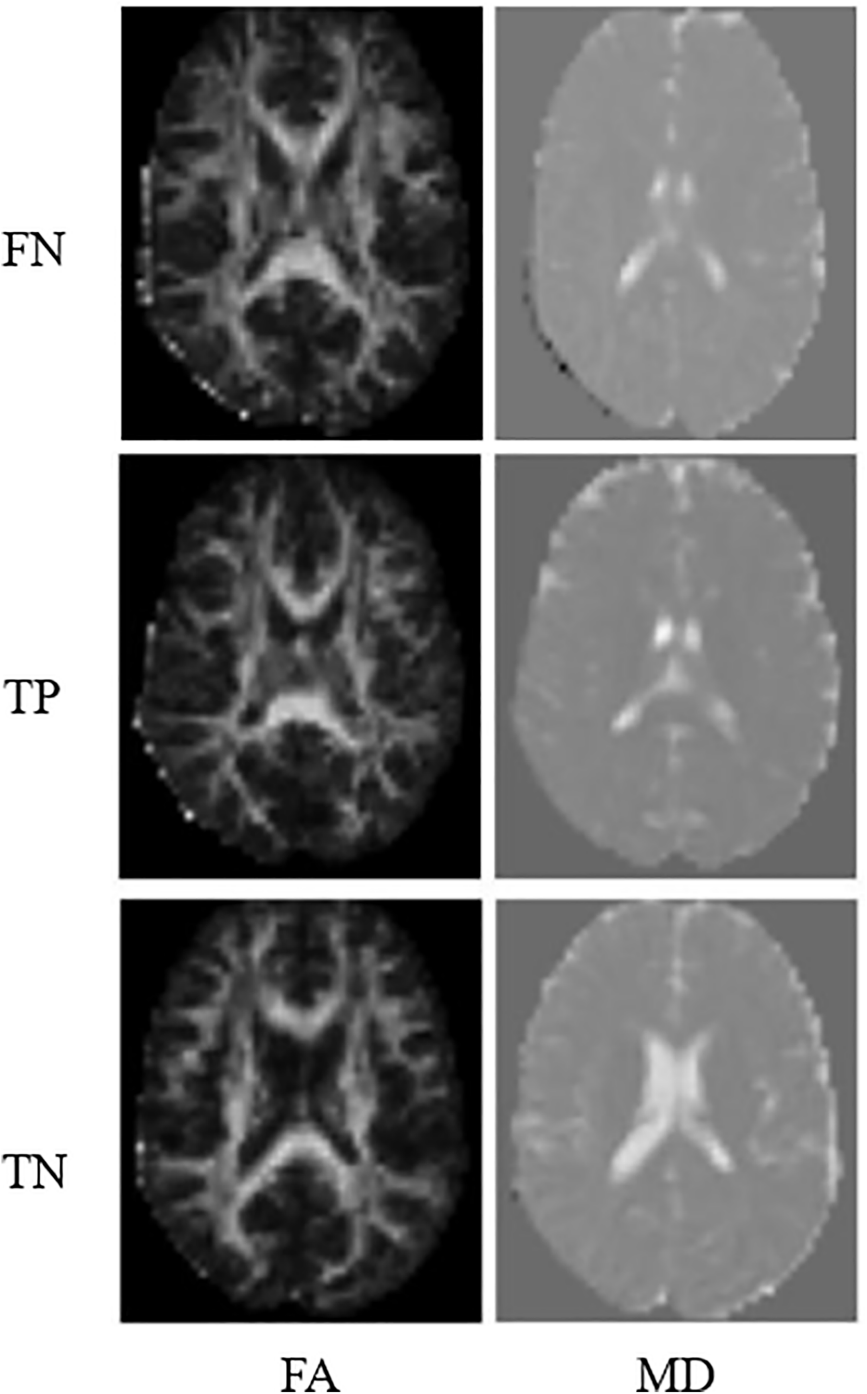
Case examples of incorrect and correct classification from the best SVM model studied.

The overall results with this approach were not good. Among the SVM models analysed, the classifier that best balanced the metric values was the linear kernel model, for the two-feature input vector. This model obtained 100% precision, 81.8% sensitivity, 100% specificity, and 91.3% accuracy. Fig 5 shows the FA and MD maps of one of the cases classified as false negative (FN), a true positive (TP) ASD patient, and another true negative (TN) patient with the best model.

**Fig 6:**
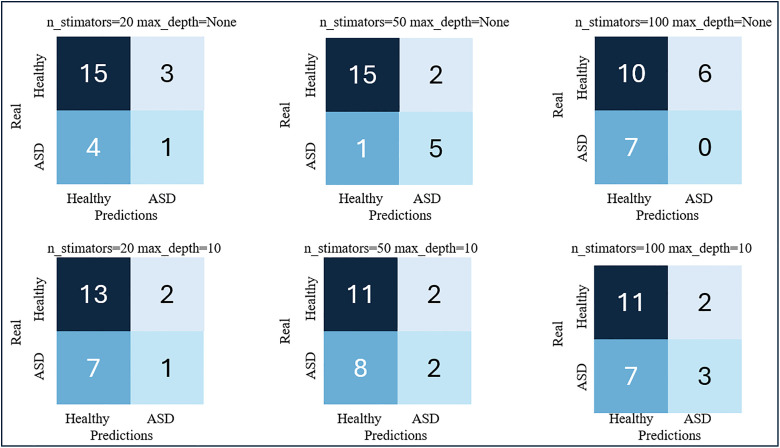
RF results for a feature vector composed on the four DTI metrics: FA, MD, RD and AD.

**Fig 7:**
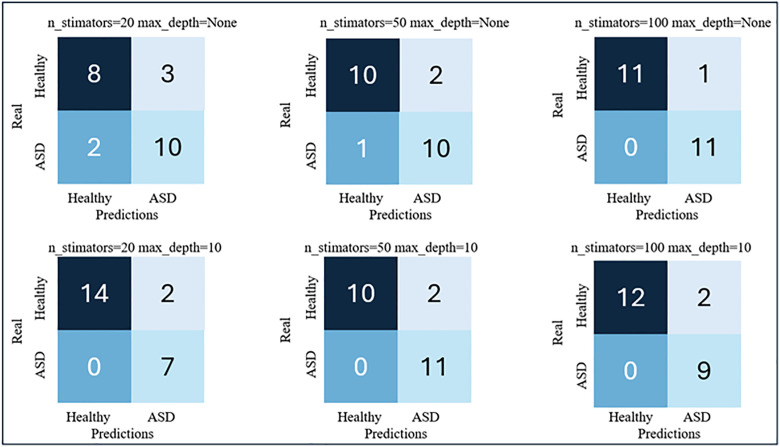
RF results for a feature vector composed on the two DTI metrics: FA and MD.

Figs 6 and 7 show the results obtained with the RF models for the 4-feature (FA, MD, RD and AD) and 2-feature (FA and MD) input vectors, respectively.

**Fig 8:**
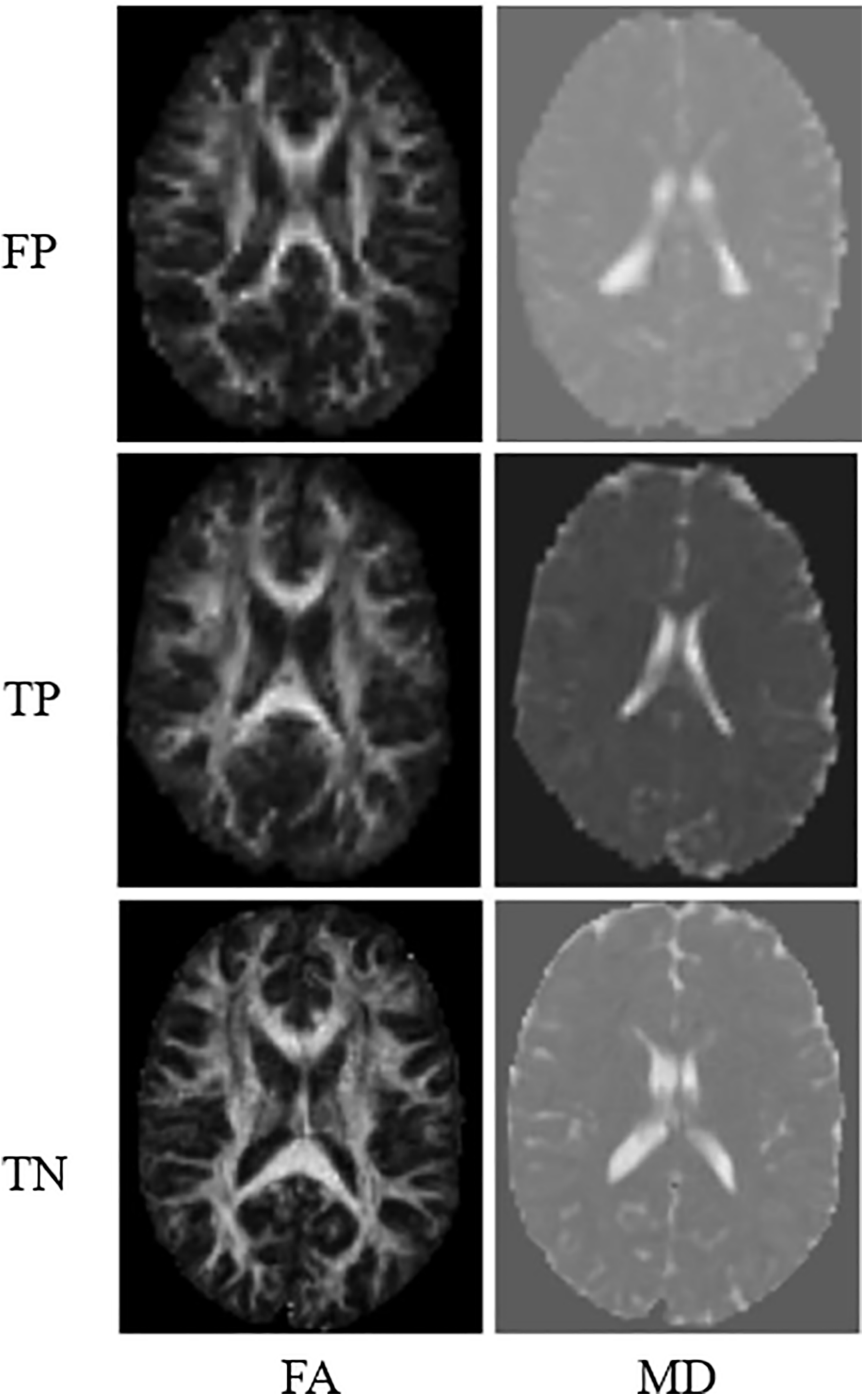
Examples of incorrect and correct classification from the best RF model studied.

We observed that the performance of this type of classification model was better than that obtained with previous SVM models. The best results were obtained for input vectors from two features. The best-performing model was the one trained by initializing with 100 trees without any depth restrictions. This is the model that presents the highest sensitivity value, which is precisely the most important metric in the opinion of medical specialists, while the rest of the metrics also maintain good results. For this model, the Precision was 91.67%, 100% sensitivity, 91.67% specificity, and 95.65% accuracy. Fig 8 shows the FA and MD maps of one of the cases classified as FP, a TP ASD patient, and another TN with the best model.

Comparing the two best models (one from each classifier), it can be seen that the chosen RF model shows high stability across all metrics (it obtained only one FP in validation) and also has the highest sensitivity for detecting ASD, which is highly desirable in the opinion of medical specialists. The SVM model, on the other hand, had the highest specificity, meaning it detected all healthy individuals very well, but it presented two false negatives in validation. Both models underwent external testing.

Note that, regarding the FA maps, the TPs present fewer bright pixels compared to the TNs, which means lower FA in some brain areas, and a loss of directionality in water diffusion, suggesting white matter involvement. However, in the MD maps, no notable differences are seen between ASD and healthy individuals with the naked eye, but the AI models are able to classify the images in ASD and healthy individuals, demonstrating the potential of ML for classification tasks from DTI images.

**Fig 9:**
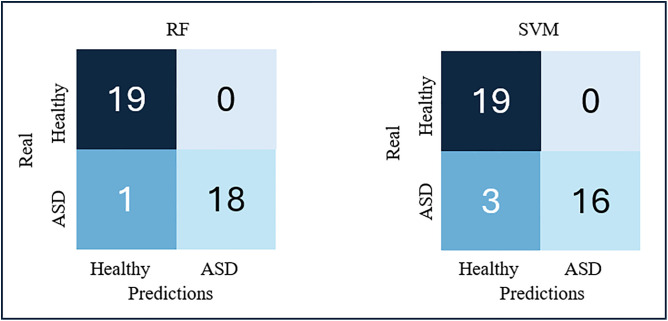
Confusion matrices of the best-rated models from the external test.

The results of the external test for the best performing RF and SVM models are shown in the confusion matrices in Fig 9. Table 3 summarizes the metrics corresponding to the results of this test for both models, which allow us to evaluate whether there is generalization power in these models.

**Table 3:** Performance metrics of the best models from the external test.

Model	Precision (%)	Sensitivity (%)	Specificity (%)	Accuracy (%)
RF	100	94.73	100	97.37
SVM	100	84.21	100	92.10

The calculated metrics show, during the external test, similar results to those of the validation tests of each model, so it can be stated that both have generalization power to detect ASD with data from diverse sources. When a model has training biases, the results of the metrics in an external test usually fall below 50% of good classification or are lower than those of validation, evidencing that there is overfitting of the model to the training data.

Overall, the RF model metrics were superior to those of the SVM model, so it was chosen as the best performing model. These results indicate that data preprocessing was effective in reducing class overlap [33,35,38–41]. They also indicate that the choice of features to consider in the classifiers has been adequate [12,42], contributing to achieving good performance with data from different sources, not previously analyzed by the model.

When analyzing the characteristics obtained in each region, alterations in the values corresponding to patients with ASD were observed compared to those corresponding to healthy individuals in most of the selected regions.

The average FA and MD values were calculated for the autistic patients and healthy individuals who were part of the training and validation set in each of the selected areas in the images. For each area, we performed a Student’s t-test for the FA and MD characteristics after analyzing the histograms, to determine whether there were significant differences in the values of these metrics between cases with ASD and healthy individuals. The results of this analysis are shown in Table 4 (see corresponding regions in the JHU ICBM-DTI-81 WM atlas in Table 2).

**Table 4:** Difference in average FA and MD values in ASD patients compared to healthy individuals.

Region	$\overset{―}{FA}$			$\overset{―}{MD}\cdot {\text{1}\text{0}}^{-\text{3}}$ mm^2^/s		
	ASD	Control	*p-values*	ASD	Control	*p-values*
Corpus callosum	0.512	0.538	0.490	0.95	0.90	0.294
Cerebral peduncle	0.561	0.577	0.714	0.92	0.89	0.000
Anterior and posterior branches of the internal capsule	0.500	0.512	0.462	0.76	0.73	0.000
Retrolenticular internal capsule	0.503	0.526	0.579	0.84	0.80	0.004
Anterior and superior corona radiata	0.421	0.430	0.000	0.78	0.76	0.027
Inferior longitudinal fascicles	0.458	0.472	0.378	0.87	0.84	0.002
External capsule	0.336	0.347	0.356	0.79	0.78	0.000
Cingulum	0.317	0.331	0.031	0.85	0.83	0.121
Superior longitudinal fascicles	0.413	0.428	0.050	0.77	0.75	0.043

In all regions analyzed, mean FA values are higher in healthy individuals than in patients with ASD, while mean MD values are higher in autistic patients than in healthy individuals, which is consistent with the scientific literature [12,14]; Shukla et al., 2010).

In healthy individuals, higher FA values indicate that water has greater directionality, while lower MD values indicate that water diffuses less in all directions. However, in patients with ASD, lower FA values indicate that water has less directionality. Increasing MD values indicate that water diffuses more freely in different directions. This suggests that, in patients with ASD, water diffusion was less anisotropic than in healthy individuals. That is, white matter fibers are less organized than in normal subjects. Therefore, water molecules can diffuse more freely and in more directions, unlike in healthy subjects, where these molecules are more directed. This means that, in individuals with ASD, there is greater white matter degeneration and impaired connectivity between different brain areas, while in healthy individuals, white matter is more organized.

Regarding the regions analyzed, FA values presented statistically significant differences between autistic and healthy subjects in the anterior and superior corona radiata, the cingulum, and the superior longitudinal fasciculi. Furthermore, MD values presented statistically significant differences between autistic and healthy subjects in all regions analyzed, except for the corpus callosum and the cingulum.

Therefore, the corpus callosum is the only region analyzed in which no significant differences were observed in the values of either of the two metrics that were key in this experiment. It was therefore concluded that it was not a statistically significantly different region between ASD and healthy subjects, unlike the research presented in [22], where it was one of the most statistically significant regions. The remaining regions showed significant differences in at least one of the two metrics.

The most statistically significant regions in the classification were considered to be those that showed significant differences in both characteristics. These regions are the anterior and superior corona radiata, the cingulum, and the superior longitudinal fasciculi, which is consistent with the research presented in [22] and [28].

### Explainability of feature reduction outcome

**Fig 10:**
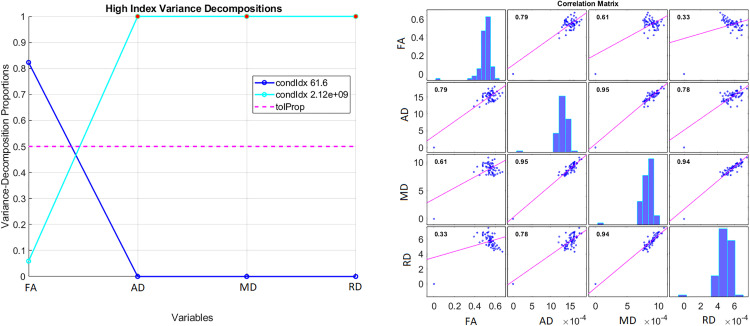
Results from running the Belsley collinearity diagnostic test for the four diffusion features in the ROI 3 for the controls. The condition indices (condIdx) identify the number and strength of any near dependencies between the variables. All condition indices are bounded between one and the condition number, which is an overall diagnostic for detecting collinearity. The variance-decomposition proportions identify groups of variates involved in near dependencies, and the extent to which the dependencies degrade the regression. The cross-correlation matrix between the four diffusion variables is shown on the right hand side panel.

Models that only used FA and MD yielded superior performance than those that included the four features, namely FA, MD, RD and AD. This can be explained by the high correlation among the diffusion variables as they are derived from the same values of the diffusion tensor. For example, MD is the average of the three eigenvalues of the diffusion tensor while RD is the average of the two smaller eigenvalues. The inclusion of highly correlated features adds redundancy to the identification of discriminative patterns and increases the risk of overfitting, especially in small samples like the one used here. By only using FA and MD our models use the most discriminative information and reduce their complexity, translating in better metrics of sensitivity and specificity. We calculated the variance inflation factor (VIF), computed Belsley collinearity diagnostics, and plotted the correlations between FA, MD, RD and AD to explain these results and demonstrate our rationale. Table 5 shows the average adjusted R^2^ value (VIF = 1/ (1 - R^2^)) from the regression of each diffusion variable using the rest as predictors. As R^2^ approaches 1, the VIF approaches infinity, indicating multicollinearity. Fig 10 shows an example of running the Belsley collinearity diagnostics between the four diffusion features in the ROI 3 for the controls. Similar results were obtained for all the rest of the ROIs in both groups (i.e., control and ASD).

**Table 5:** Average adjusted R^2^ value from the regression of each diffusion variable using the rest as predictors, to determine the VIF.

Region	Code ICBM-DTI-81	ASD	Controls
Corpus callosum	3,4,5	0.969	0.952
Cerebral peduncle	15,16	0.971	0.969
Anterior and posterior branches of the internal capsule	17,18,19,20	0.984	0.954
Retrolenticular internal capsule	21,22	0.948	0.957
Anterior and superior corona radiata	23,24,25,26	0.958	0.933
Inferior longitudinal fascicles	31,32	0.980	0.954
External capsule	33,34	0.992	0.976
Cingulum	35,36,37,38	0.992	0.969
Superior longitudinal fasciculi	41,42	0.990	0.956

### Risk of bias and generalizability

We analyzed demographic and clinical variables from all subsets of data from the three contributing centres. Table 6 shows the descriptive statistics (i.e., average age (standard deviation), performance IQ, verbal IQ and full IQ, and the sex and handedness information) for each subgroup. ASD and control groups for the training and validation sets were balanced in terms of age and handedness (proportions) but not in sex splits. In the external test set, all (i.e., ASD and controls) were left handed male. Compared with the training and validation set, the individuals in the external testing set were older. The differences in IQ between ASD and controls in the training and validation set were smaller on average than between ASD and controls in the external test set, where the controls had on average 9 units higher IQ scores than the ASD group. The heterogeneity of the samples from different sites and with different demographic characteristics confers confidence in the generalizability of the proposed model and in the validity of the analyses presented.

**Table 6:** Demographic variables and IQ information per subgroup.

Subgroup	Age (years)	Sex	Handed-ness	Performance IQ	Verbal IQ	Full IQ
T&V ASD (n = 68)	11.07 (5.38)	M: 57 F: 11	RH: 29 LH: 27 MH: 7	103.29 (18.96)	101.67 (18.32)	101.94 (17.01)
T&V Controls (n = 44)	11,74 (3.80)	M: 41 F: 3	RH: 24 LH: 18 MH: 1	105.82 (16.04)	110.50 (12.95)	108.75 (14.44)
External Test ASD (n = 19)	14.67 (3,27)	M: 19 F: 0	RH: 0 LH: 19 MH: 0	108.37 (13.86)	109.63 (14.89)	110.37 (14.01)
External Test Controls (n = 19)	15.62 (3.14)	M: 19 F: 0	RH: 0 LH: 19 MH: 0	116.10 (11.88)	118.16 (15.96)	119.26 (13.29)
Excluded ASD (n = 42)	8.81 (3.25)	M: 38 F: 4	RH: 16 LH: 12 MH: 6	103.38 (17.46)	103.22 (14.34)	103.02 (14.98)
Excluded Controls (n = 13)	10.09 (2.95)	M: 12 F: 1	RH: 10 LH: 2 MH: 0	112.61 (11.52)	112.61 (17.64)	113.61 (14.01)

Legend: T&V: training and validation set, ASD: autism spectrum disorder, RH: right handed, LH: left handed, MH: mixed handed

Due to the lack of the bval/bvec data or incomplete/unusable images, 55 individuals (42 ASD and 13 controls) from the three contributing centres were excluded. They were much younger on average than those who contributed to the training, validation and testing of the models. Given that only four were from the TCD, used as external set, their putative inclusion would have made wider the age disparity between the individuals who contributed information to develop the model and those who contributed in the external testing of the model. Also, as the differences in IQ between ASD and control groups in this excluded dataset were large, their putative inclusion would have made the training/validation set closer to the external dataset in this regard, which would have diminished confidence in the generalizability of the model. It is worth noting that in the excluded subsamples and in the subsamples used in the development of the models there were more males than females, so the putative inclusion of the excluded data would not have facilitated a more balanced data in terms of sex splits either for the development of the models, and rather would have increased the sex imbalance in the development sample in favor of males, perhaps deeming the testing set (which only has data from males) even less relevant in terms of showing the generalizability capacity of the model proposed.

We also generated box plots of the clinical characteristics of the subgroups of individuals who contributed data to the training, validation, and external testing of the models (Supplementary material). As can be appreciated, although across all tests there are differences between the responses from ASD and controls, the means, medians and distributions, especially from the scores from all tests that formed part of the child behavior checklist (ages 6–18), largely differ between the training/validation and the external test groups (see S3 Fig), strengthening the confidence in the capacity of the model proposed to perform well in an unseen population.

## Discussion

Detecting ASD is currently a challenge. Although descriptive screening methods [6,43–45] help detect ASD, this cannot always be achieved early. These methods pose challenges for specialists, as they involve lengthy and time-consuming questionnaires for patients and family members. However, they are the methods that have prevailed for years for clinical detection. In most cases, they are administered by psychologists, psychiatrists, or neurologists and require a lengthy follow-up observation process for definitive evaluation [46].

Furthermore, despite the numerous advantages that morphological MRI images offer in identifying ASD, their quality significantly impacts the diagnostic accuracy of clinicians [47]. Furthermore, MRI data from individuals with ASD are obtained through multiple slices and different protocols. As a result, considerable time is also required to thoroughly examine the images. Professionals must be extremely careful, as eye strain can lead to misdiagnoses [48], often necessitating the need for second opinions, which can delay diagnosis. For all these reasons, early detection of ASD is very complex.

DTI images offer a unique view of brain structure and connectivity at the microscopic level, allowing for an assessment of anatomical connections and evaluating brain connectivity based on the organization and integrity of white matter, which is essential for communication between different brain areas [49].

For these reasons, the potential of the DTI-based RF model obtained is highlighted, as it would allow for faster diagnosis and help prevent eye strain, making it a useful support tool for specialists.

The model obtained classifies better for a two-feature input vector (FA and MD). This may be due to the high heterogeneity of the DB used. FA and MD have been shown to be powerful biomarkers after controlling for age, sex, or IQ [14]. However, RD and AD features have been much less studied, and their values may be biased by some of these elements, aspects that warrant future study.

Regarding computational cost, the model training stage took 5 seconds per model with the computational capabilities used. However, the image preprocessing stage resulted in a computational cost of between 1.5 and 2 hours per image, making this stage unfeasible in clinical practice. Images must be preprocessed under high computational performance conditions, for example, using a computer cluster in an institution with these facilities.

Comparing the results obtained with those of previous studies in the scientific literature is not a simple task. This is because systems trained using different databases, calculating different metrics, and employing diverse classification methods are published. To conduct a proper comparison, a review of two recent studies was conducted, which used the same database as in this work (ABIDE II).

In [39], a logistic regression model was trained to calculate a Euclidean distance-based correlation matrix from five DTI features (FA, MD, AD, RD, and anisotropy mode). In this study, it was observed that features obtained from the anterior limb of the left internal capsule to the right superior corona radiata regions contributed most to ASD classification.

In [49], several models were trained, including SVM, RF, logistic regression, neural networks, among others. These classifiers were trained using statistical features obtained from six DTI features (FA, MD, $\lambda_{\text{1}}$, $\lambda_{\text{2}}$, $\lambda_{\text{3}}$, and tensor skewness). The best trained model was the linear SVM. With respect to these studies, the results of the model obtained in the present work are superior to those reported in [28]. Although the accuracy of the model presented in [20,21] is higher than the value obtained in this study, this model only showed validation results, without external testing, so it is not known whether it has generalization power for data different from those used in training.

Although a comparison between studies conducted under different conditions is not appropriate, the comparative analysis of the results can be considered a reference framework in relation to currently accepted international standards. Thus, for example, in study [29], the DB USC Multimodal Connectivity Database was used, consisting of diffusion images from 51 patients with ASD and 41 healthy controls. Metrics based on connectivity matrices were calculated. The algorithms used were SVM and Linear Discriminant Analysis. The best-performing model showed a sensitivity of 81.94% and a specificity of 70.42%.

On the other hand, in [27], the DB used consisted of two sets of diffusion images obtained from the National Database for Autism Research (NDAR), and the SVM algorithm with a vector was used, obtaining a sensitivity of 72% and a specificity of 70%.

These comparisons confirm that the model obtained in this research is within the range of internationally accepted models and presents superior results to some proposed in previous studies. The relatively small sample size used could be considered a limitation of this work. A recent work [50] has proposed an approach that integrates the power of generative adversarial networks (GANs) and Deep Q-Learning to augment limited datasets (namely ABIDE I and II), and evaluates it through the task of identifying ASD using resting state functional MRI. The best performance across the 10 classifiers evaluated by Zhou and colleagues reported the combination of the proposed approach and InfoGAN achieved an accuracy of 87.30%, a sensitivity of 91.60%, and a specificity of 86.49%, all below the figures reported here, but above those achieved without data augmentation or with other data augmentation methods. Future work could use such approach to generate a large sample and re-evaluate the best configuration proposed here.

The success of the best model proposed in this work shows that the preprocessing steps performed on the images, the classification models selected, and the choice of white matter areas to carry out an analysis based on regions of interest, rather than using the entire brain or a specific region, were superior to the approaches followed to date in research [22,27,28,51]. However, our model still can be improved. The application of more systematic hyperparameter tuning strategies like GridSearchCV or RandomSearchCV in future works could optimize the model performance and the stability of the results. Also, further validation in other samples is now needed, involving larger number of age and sex-matched ASD/control cases, individuals from underrepresented socio-economic groups and diverse ethnicity with images from different sites and acquired with different protocols.

A notable aspect is the practical utility of having employed ML methods that do not perform deep learning, such as those required in convolutional neural networks [51,52]. However, these systems that require large amount of data for training are vulnerable to the different sources of bias in the data available for their development. This is one of the most widely recognized challenges to overcome in the translation of the CAD systems developed using research data to clinical practice [53]. The generalization capacity of a ML model with a reduced number of data is an essential element, especially for the medical sector. When handling limited data sets, it is crucial to extract the most relevant and significant information from them, which allows training more robust and generalizable classification models [54], eliminating noise and redundancy while delivering a fairer response.

## Conclusions

This research demonstrated that a ML model incorporating a RF classifier is effective in detecting ASD from DTI, even when trained with a relatively low amount of data.

The proposed model performed well in terms of computational effectiveness and efficiency and demonstrated generalization power, obtaining equivalent results in external testing and validation.

The superior longitudinal fascicles, the anterior and superior corona radiata, and the cingulum were the most significant regions in the classification of ASD cases and healthy individuals, while the corpus callosum was the only region that did not show significant differences.

The sensitivity and specificity values obtained by the two best models chosen for validation exceed 90%, being on the order of or exceeding those obtained by international CAD systems. A prospective validation is now needed.

## Supporting information

S1 Fig Box plots of the raw and T-score results from the Social Responsiveness Scale applied to the individuals that contributed data to the study, grouped by ASD and controls in the training/validation and external test subsets. Data source: ABIDE II (https://fcon_1000.projects.nitrc.org/indi/abide/abide_II.html).
(TIF)

S2 Fig Box plots of the scores from the Repetitive Behaviors Scale (Revised 6) applied to the individuals that contributed data to the study, grouped by ASD and controls in the training/validation and external test subsets. Data from the Trinity Centre for Health Sciences (TCD), who was used as external validation set, did not contain these results for controls. Data source: ABIDE II (https://fcon_1000.projects.nitrc.org/indi/abide/abide_II.html).
(TIF)

S3 Fig Box plots of the T-scores from the Child Behavior Checklist for ages 6–18, applied to the individuals that contributed data to the study, grouped by ASD and controls in the training/validation and external test subsets. Data source: ABIDE II (https://fcon_1000.projects.nitrc.org/indi/abide/abide_II.html).
(TIF)
