# Supplementary figures and images for: Autism spectrum disorder detection using diffusion tensor imaging and machine learning
Source: PLOS Digit Health. 2025 Dec 23;4(12):e0001155. doi: 10.1371/journal.pdig.0001155 (PMC12725754; doi:10.1371/journal.pdig.0001155)

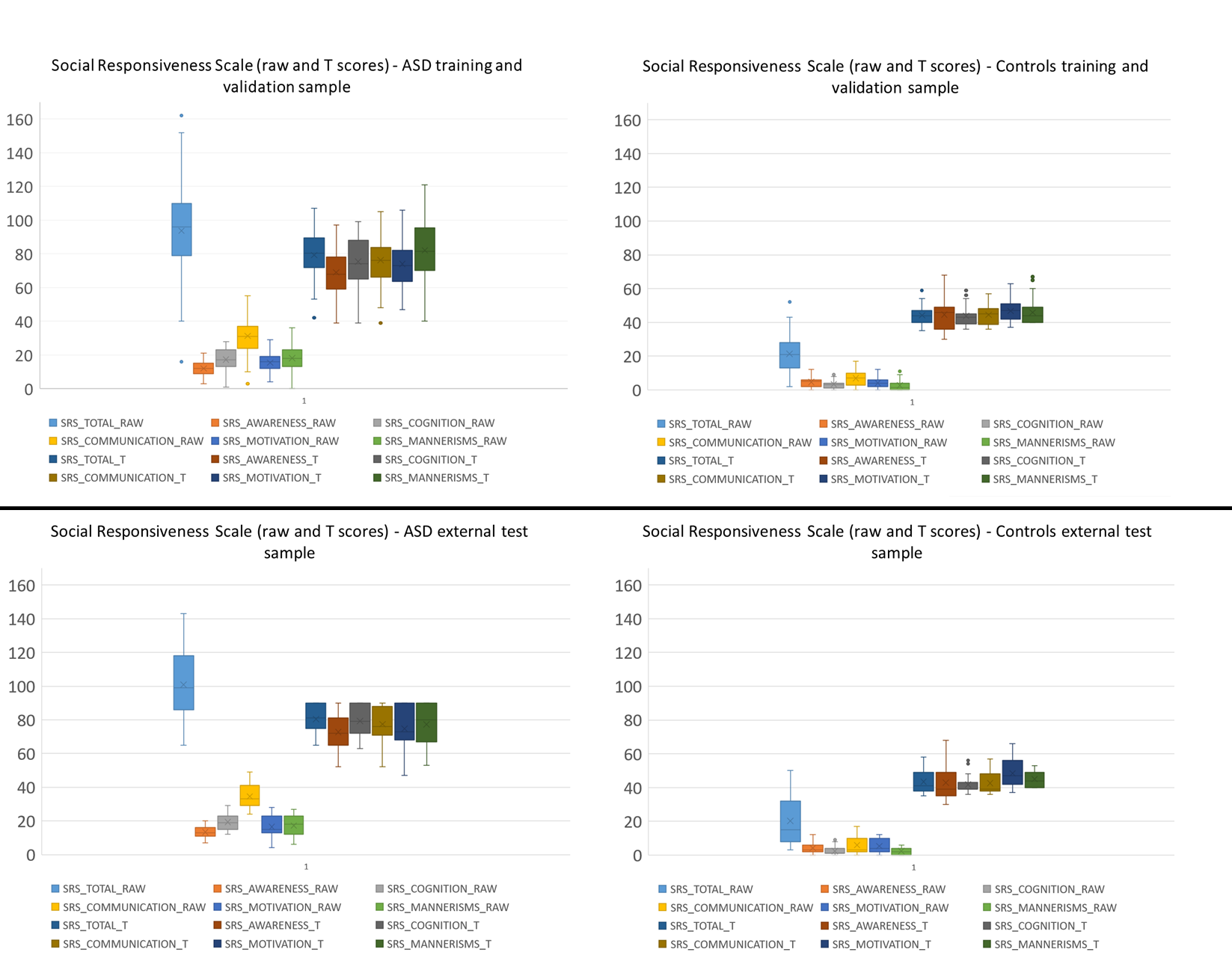

Supplement: S1 Fig — Data source: ABIDE II (https://fcon_1000.projects.nitrc.org/indi/abide/abide_II.html). (TIF) [file pdig.0001155.s001.tif]

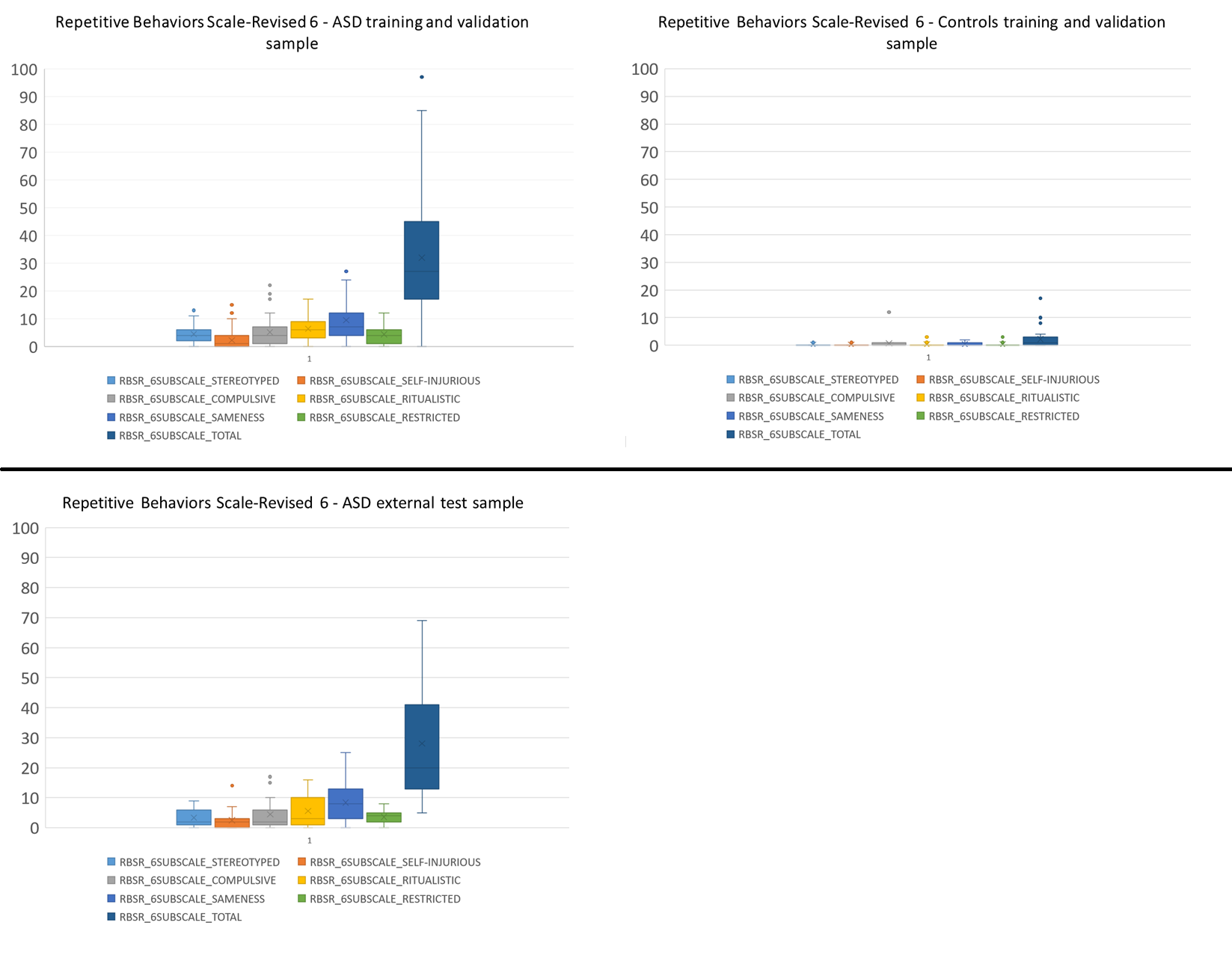

Supplement: S2 Fig — Data from the Trinity Centre for Health Sciences (TCD), who was used as external validation set, did not contain these results for controls. Data source: ABIDE II (https://fcon_1000.projects.nitrc.org/indi/abide/abide_II.html). (TIF) [file pdig.0001155.s002.tif]

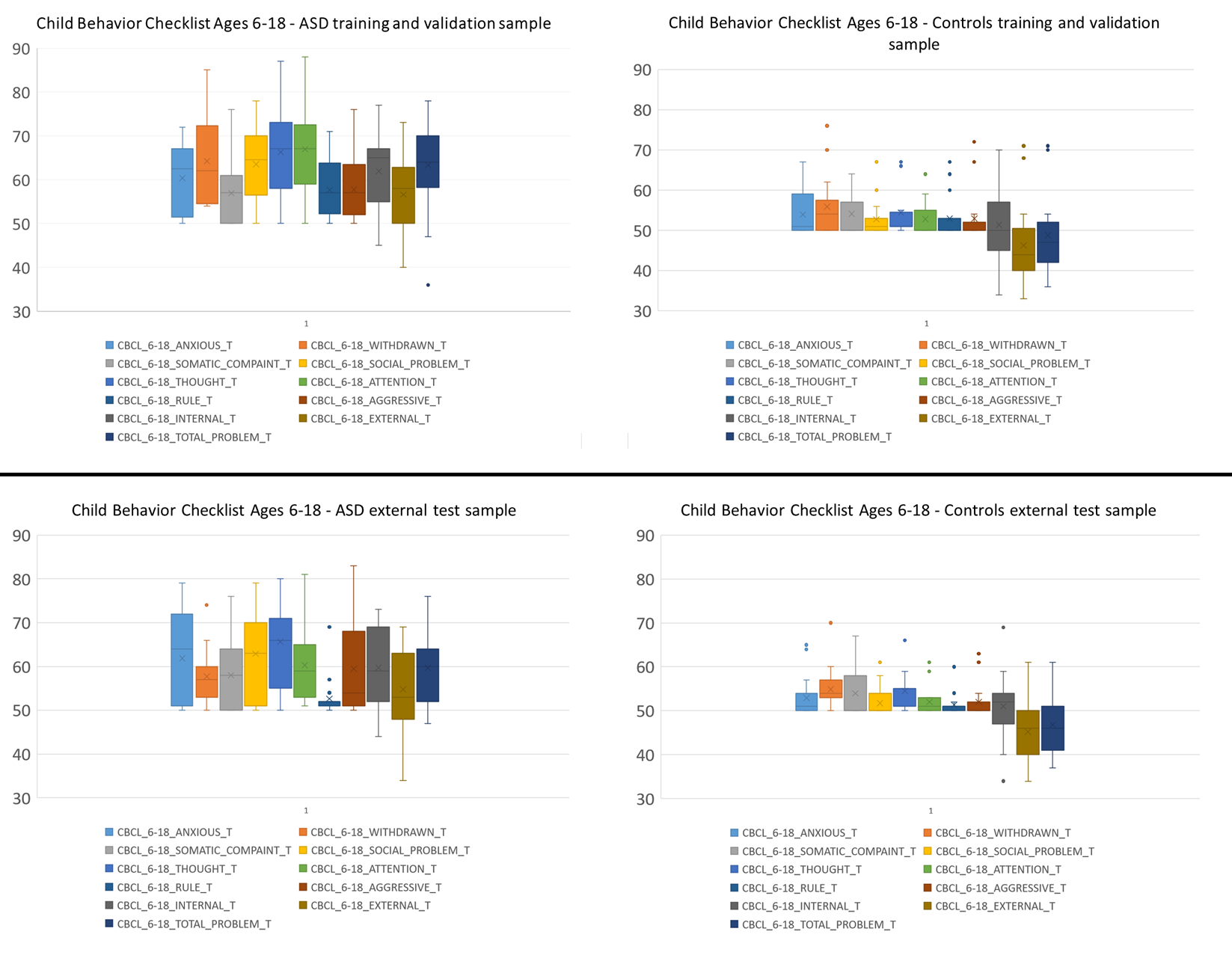

Supplement: S3 Fig — Data source: ABIDE II (https://fcon_1000.projects.nitrc.org/indi/abide/abide_II.html). (TIF) [file pdig.0001155.s003.tif]
